# FABIAN-variant: predicting the effects of DNA variants on transcription factor binding

**DOI:** 10.1093/nar/gkac393

**Published:** 2022-05-26

**Authors:** Robin Steinhaus, Peter N Robinson, Dominik Seelow

**Affiliations:** Exploratory Diagnostic Sciences, Berlin Institute of Health, 10117 Berlin, Germany; Institute of Medical Genetics and Human Genetics, Charité – Universitätsmedizin Berlin, corporate member of Freie Universität Berlin and Humboldt-Universität zu Berlin, 13353 Berlin, Germany; The Jackson Laboratory for Genomic Medicine, Farmington, CT 06030, USA; Institute for Systems Genomics, University of Connecticut, Farmington, CT 06030, USA; Exploratory Diagnostic Sciences, Berlin Institute of Health, 10117 Berlin, Germany; Institute of Medical Genetics and Human Genetics, Charité – Universitätsmedizin Berlin, corporate member of Freie Universität Berlin and Humboldt-Universität zu Berlin, 13353 Berlin, Germany

## Abstract

While great advances in predicting the effects of coding variants have been made, the assessment of non-coding variants remains challenging. This is especially problematic for variants within promoter regions which can lead to over-expression of a gene or reduce or even abolish its expression. The binding of transcription factors to the DNA can be predicted using position weight matrices (PWMs). More recently, transcription factor flexible models (TFFMs) have been introduced and shown to be more accurate than PWMs. TFFMs are based on hidden Markov models and can account for complex positional dependencies. Our new web-based application FABIAN-variant uses 1224 TFFMs and 3790 PWMs to predict whether and to which degree DNA variants affect the binding of 1387 different human transcription factors. For each variant and transcription factor, the software combines the results of different models for a final prediction of the resulting binding-affinity change. The software is written in C++ for speed but variants can be entered through a web interface. Alternatively, a VCF file can be uploaded to assess variants identified by high-throughput sequencing. The search can be restricted to variants in the vicinity of candidate genes. FABIAN-variant is available freely at https://www.genecascade.org/fabian/.

## Graphical Abstract

**Graphical Abstract:**
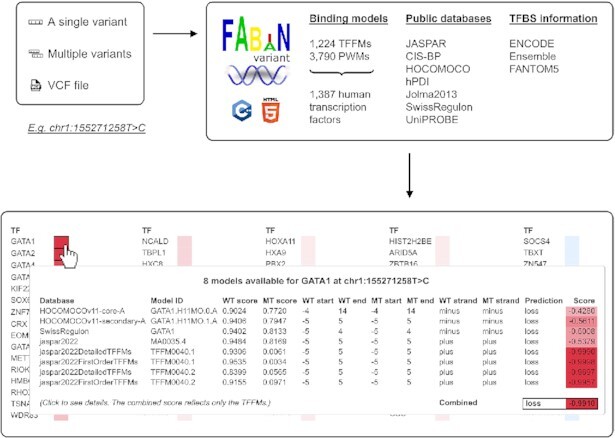
FABIAN-variant: analysis of a promoter variant that has been reported to disable binding of the erythroid transcription factor GATA1 (chr1:155271258T>C).

## INTRODUCTION

Any human individual harbours about 4 million short variants (single nucleotide variants and short indels) in their genome compared to the reference genome (1). Many computer programs are available for assessing the disease-causing potential of variants in coding regions (e.g. MutationTaster (2), PolyPhen (3), and SIFT (4)), but coding regions make up only about 1.5 % of the human genome. Much less is known about the effect of variants in the remaining non-coding DNA, and tools aiming at the prediction of such variants (e.g. CADD (5), Genomiser (6), and RegulationSpotter (7)) are hampered by the lack of known disease mutations outside of protein-coding genes as training cases.

Variants in regions essential for gene expression (especially promoters and enhancers) can alter the binding affinity of transcription factors, leading to up- or down-regulation of transcriptional activity (8). This may result in non-expression or severe under-expression of a gene, the subsequent loss of the encoded protein (null mutation), and lead to disease (9–11).

The prediction of transcription factor binding sites (TFBSs) presents an ongoing challenge in computational biology (12,13). The standard method for assessing the binding affinity of a transcription factor to DNA in silico is to compare the DNA bases with a position weight matrix (PWM) specific for the transcription factor. A PWM model is obtained from an assumed common binding motif by counting adenine, cytosine, guanine, and thymine bases at each position in experimentally-confirmed binding sites for a transcription factor. Many thousands of PWM profiles have been published in open-access databases (e.g. JASPAR (14), HOCOMOCO (15), and SwissRegulon (16)).

PWMs are relatively simple models that ignore the positional dependencies that have been repeatedly observed in TFBSs (17–20). More advanced models have in many cases been shown to give better results in identifying experimentally verified binding sites (21–23). A number of alternative modelling approaches have been proposed, several of which attempt to integrate dependencies between adjacent and/or distant positions. These include the binding energy model (BEM) (24), dinucleotide weight matrices (DWMs) (25), and transcription factor flexible models (TFFMs) (22). Among these, TFFMs have been gaining visibility since their inclusion in the JASPAR database for transcription factors, which includes >1000 human TFFMs in its current release (14). TFFMs are based on hidden Markov models (HMMs) and can account for complex positional dependencies as well as variable length nucleotide patterns. TFFM motifs are defined in terms of HMM states, transitions between states, initials, and emissions. Two types of TFFMs are commonly used and supported in FABIAN-variant: In first-order TFFMs, each position within a TFBS is represented by a HMM state emitting a nucleotide with probabilities dependent on the nucleotide found at the previous position. In detailed TFFMs, each HMM state in the first-order HMM is decomposed into four states (one per nucleotide) and transition probabilities reflect the emission probabilities of the first-order HMM (22). Like PWMs, TFFMs are derived from experimentally verified binding sites. Unlike PWMs, TFFMs cannot be evaluated using standard mathematical operations, but require dedicated algorithms for HMMs. Although several tools for evaluating PWMs exist (e.g. FIMO (26), motifbreakeR (27), and Pscan (28)), we have only found one other web application that works with TFFMs (TFBSPred (29)). However, TFBSPred does not provide a mechanism for evaluating DNA variants.

This article introduces a new user-friendly web application for predicting the effects of DNA variants on transcription factor binding. FABIAN-variant offers 1224 TFFMs and 3790 PWMs from different databases for 1387 different human transcription factors. It has different modes for analysing single variants, lists of variants, or up to 10 000 variants from a VCF file. For each variant and transcription factor, FABIAN-variant evaluates available models in the ‘wild-type’ and variant sequence and returns a combined score indicating whether or not and to which degree transcription factor binding may be affected. The backend is written in C++ for speed and most types of analysis are completed in just a few seconds. For VCF-based analyses, users can choose to be notified by email once the run completes. Results are visualised in the browser and can also be downloaded. Various filters for regions, genes, variants, and transcription factors are implemented (e.g. search in promoter regions of candidate genes, search with TFFMs only). Genome builds GRCh37 (hg19) and GRCh38 (hg38) are supported. FABIAN-variant is free and open to all users without login requirement.

## SOFTWARE/BACKEND

### Evaluation of TFFMs and PWMs

Different models for human TFBSs (TFFMs and PWMs) were downloaded from various data sources (Table 1) and imported into FABIAN-variant.

**Table 1.** Data sources included in FABIAN-variant

Source	Data	URL	Reference
JASPAR 2022	612 detailed TFFMs	https://jaspar.genereg.net/	(14)
JASPAR 2022	612 first-order TFFMs	https://jaspar.genereg.net/	(14)
JASPAR 2022	877 PWMs	https://jaspar.genereg.net/	(14)
MotifDb 1.36.0	*	https://doi.org/10.18129/B9.bioc.MotifDb	(41)
CIS-BP 1.02	313 PWMs	http://cisbp.ccbr.utoronto.ca/	(42)
HOCOMOCO 11	768 PWMs	https://hocomoco11.autosome.org/	(15)
hPDI	436 PWMs	http://bioinfo.wilmer.jhu.edu/PDI/	(43)
Jolma 2013	710 PWMs	https://doi.org/10.1016/j.cell.2012.12.009	(44)
SwissRegulon	684 PWMs	https://swissregulon.unibas.ch/sr/	(16)
UniPROBE	2 PWMs	http://the_brain.bwh.harvard.edu/uniprobe/	(45)
ENCODE 3	7,374,455 TFBSs^†^	https://www.encodeproject.org/	(30)
Ensembl Regulation 102	7,808,345 TFBSs^†^	https://www.ensembl.org/	(31)
FANTOM5 SSTAR	4,987 TFBSs^†^	https://fantom.gsc.riken.jp/5/sstar/	(32)

Data included in this table and in FABIAN-variant is for human transcription factors only.

*MotifDb 1.36.0 is an annotated collection of PWM models, and we obtained all PWMs listed in this table except for JASPAR 2022 from MotifDb.

^†^Data for genome build GRCh37 is shown.

FABIAN-variant evaluates each selected TFFM and PWM model in a sliding window from −15 to +15 nucleotides around the variant location in both the reference sequence (‘wild-type’, *WT*) and the variant sequence (‘mutated’, *MT*). Both strands are considered. Then the highest scores for both sequences (0 ≤ *WT*,*MT* ≤ 1) are compared for each model. A greater *WT* score indicates a weakened binding affinity, and a greater *MT* score indicates an increased binding affinity caused by the variant. For each model, FABIAN-variant generates a joint score *S* between −1 (likely TFBS loss) and 1 (likely TFBS gain),

$$\begin{eqnarray*} S = \frac{2}{1+2^{-2F}}-1,\quad F = \left\lbrace \begin{array}{ll}-\dfrac{1 - MT + \alpha }{1 - WT + \alpha } + 1 & \quad WT >MT\\ \\ \dfrac{1 - WT + \alpha }{1 - MT + \alpha } - 1& \quad WT \le MT\\ \end{array} \right. \end{eqnarray*}$$

with pseudocount α = 0.1 to avoid zero in the denominator. We use the inverse of *WT* and *MT* in the ratio (e.g. 1 − *WT*) to account for the fact that PWM and TFFM scores correlate with the likelihood that binding is possible in the first place and the ratio is comparatively higher with small denominators. *WT*, *MT* and the joint score *S* per model are shown on the results page of FABIAN-variant.

For most transcription factors, several models (TFFMs and PWMs) exist. To obtain the combined prediction per variant per transcription factor, FABIAN-variant calculates the average of joint scores *S* of the individual models. If both TFFMs and PWMs are available, FABIAN-variant by default uses only the results from TFFMs for the combined prediction (this setting can be changed on the results page so that both types of models are included in the combined score).

### Known TFBSs

To allow the restriction to known TFBSs, we collected data from ChIP-seq experiments from ENCODE (30) and Ensembl Regulation (31), as well as from cap analysis of gene expression (CAGE) experiments from FANTOM5 (32) (Table 1). Please note that TFBSs obtained from ChIP-seq experiments are regions of several hundred bases, whereby the precise location of the actual TFBS within the region is however unknown.

We did not use Ensembl’s motif-derived predicted binding sites.

## FEATURES/FRONTEND

### Search interface

**Figure 1.**
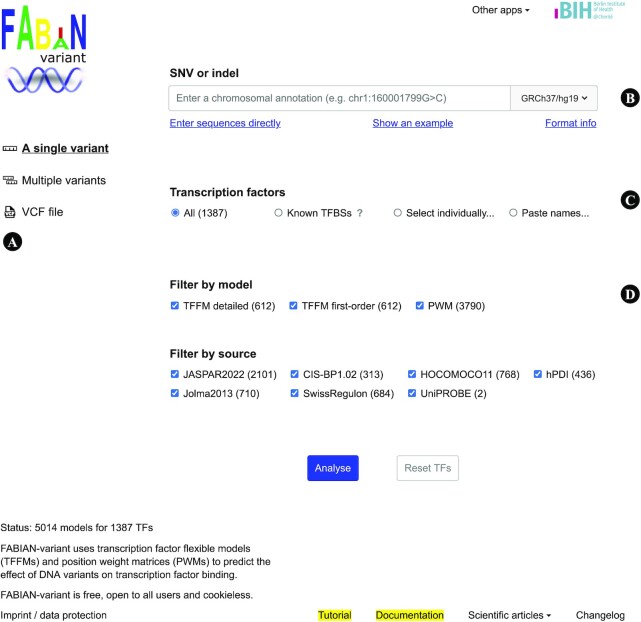
FABIAN-variant interface for a single variant. (**A**) Users can choose between a single variant, multiple variants, or a VCF file. (**B**) Input field for a chromosomal annotation of a single variant (e.g. 1:160001799G>C). Variants can also be entered as nucleotide sequences by clicking ‘Enter sequences directly’ (e.g. GGCCCTC...>TCACACT...). (**C**) ‘Known TFBSs’ searches for transcription factors known to bind at the location of the variant based on ENCODE, Ensembl, or FANTOM5 data. ‘Select individually...’ and ‘Paste names...’ open fields to submit a custom set of transcription factors. (**D**) The type of models can be restricted to TFFMs, PWMs, or data from specific sources. The numbers in parentheses update automatically and indicate the number of models included in the search based on the current input.

On the search page (Figure 1), users can select transcription factors and choose models to be included in the search. Variants can either be entered into a text field or uploaded as a VCF file. In the latter case, we provide filter options for candidate gene regions, custom genomic regions, coverage, homozygosity, and restriction to rare variants using data from gnomAD (33) and the 1000 Genomes Project (34).

Users can choose to include all 5014 models and all 1387 transcription factors or limit the search to specific factors and models. The search can be restricted to transcription factors for which there are known binding sites at the genomic location of the variant. Other options are to use only TFFMs, only PWMs, or only models from a specific database.

Results for search of a single variant are available immediately after clicking on ‘Analyse’. If all models and not >100 variants are included in the search, FABIAN-variant usually returns the results in <90 s.

### Results overview

**Figure 2.**
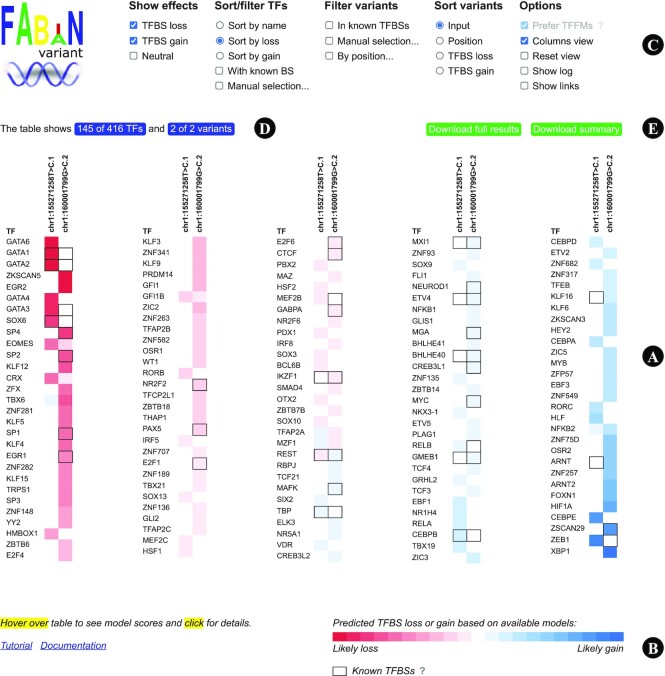
Results page for two promoter variants. (**A**) The results are divided into five sections for better readability. Variants are plotted in columns, transcription factors in rows. Coloured cells indicate the potential loss (red) or gain (blue) of a TFBS due to the variant. (**B**) Legend. Deeper shades of red or blue represent a greater loss or gain. Known TFBSs at the location of a variant are displayed with a border around the cell. Please note that the TFBSs obtained from ChIP-seq experiments are regions of several hundred bases and we do not know where within these stretches the real binding sites are located. (**C**) Users can define sorting and filters to limit the displayed data. (**D**) 145 transcription factors are currently shown in the table. The number is refreshed automatically based on active filters. (**E**) Results can be downloaded.

A sample results page for two pathogenic promoter variants is shown in Figure 2 (chr1:155271258T>C has been reported to disable binding of the erythroid transcription factor GATA1 (35) and chr1:160001799G>C to disrupt binding of SP1 (36)). Coloured cells indicate the likelihood of a loss (red) or gain (blue) of a TFBS due to the variant based on the combined prediction of different models per variant. Deeper shades of the colour represent a greater loss or gain. Moving the mouse pointer over a coloured cell reveals the individual model scores.

The results page provides access to all results for all variants, transcription factors, and models included in the search. Because the amount of results can be overwhelming for large searches, there are several sorting and filtering options at the top that can be used to reorder or hide information on the page. Changes to the options are immediately reflected in the results table. The filter options allow the user to only show transcription factors within a specified genomic region, with a predicted loss or gain of a TFBS, with a known TFBS at the location of a variant, or those which are manually selected with the mouse pointer. FABIAN-variant automatically applies pagination beyond 100 variants. We provide a download of the complete results in TSV format and a summary based on the selected filters.

### Detailed results

**Figure 3.**
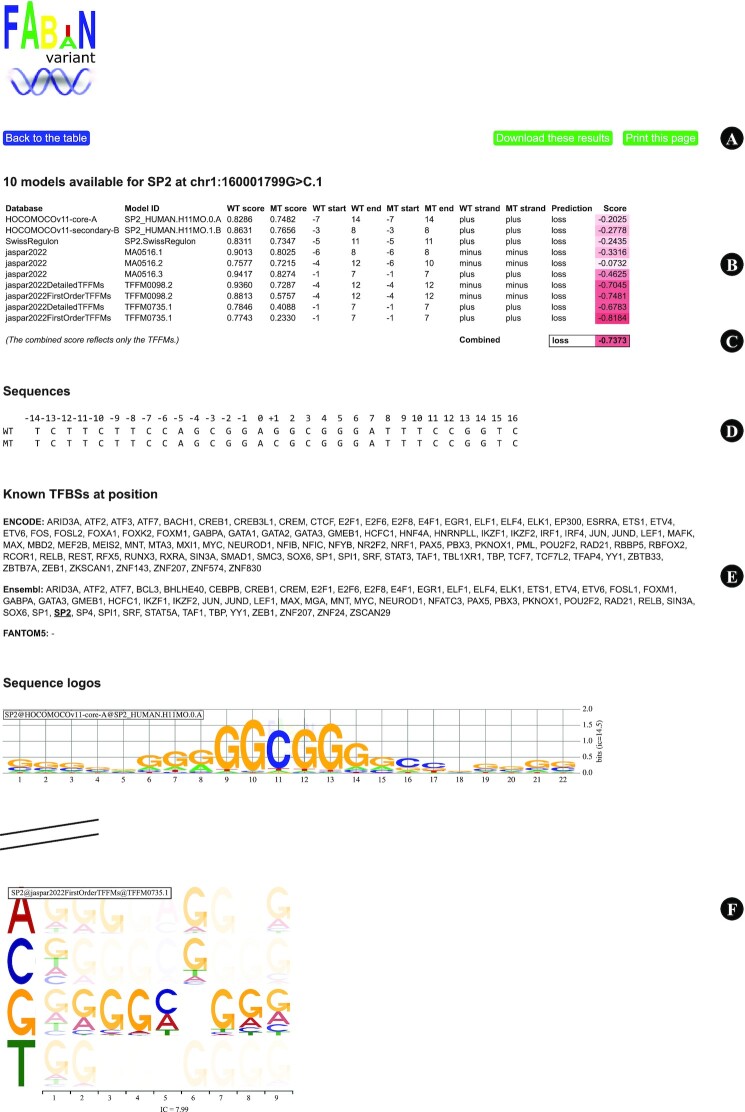
The detailed results page is shown after clicking on the corresponding cell in the results table. (**A**) Options to download or print details on this page. (**B**) Six PWMs and four TFFMs for transcription factor SP2 were evaluated for variant chr1:160001799G>C (GRCh37). Higher scores in the reference sequence (WT) than in the variant sequence (MT) indicate a possible loss of a TFBS. (**C**) The combined prediction is shown below the individual model scores. (**D**) Reference and variant sequences (variant at position +1). (**E**) A list of known TFBSs at the variant location. (**F**) Sequence logos and information content for the ten models (abridged in the Figure).

Clicking on a coloured cell brings up detailed results for an individual variant and a single transcription factor (Figure 3). The page includes the model scores, an option to print results, reference and variant sequences, a list of all known TFBSs at the variant location, as well as sequence logos for the different models.

## IMPLEMENTATION

FABIAN-variant is an acronym for FAst BInding-site ANalysis and has been optimised for computational efficiency. The FABIAN-variant web server uses Perl CGI to run the C++ backend and a PostgreSQL database. The frontend includes JavaScript and Ajax for interactivity. Job scheduling is provided by Slurm. The code does not use other third-party libraries.

Each TFFM score is computed with a custom C++ implementation of the forward-backward algorithm from the GHMM library (37). Position count matrices (PCMs) were converted to PWMs using the method described in (38) based on the background nucleotide distribution in the human genome.

## DISCUSSION

Since their inclusion in the JASPAR database for transcription factors, TFFMs are gaining visibility. FABIAN-variant is the first web application that can not only analyse variant effects with PWMs but also with TFFMs.

Because there are millions of non-coding variants in any human genome, it is not helpful to search for effects on transcription factor binding for all of them – one would drown in results. However, the search for potentially regulatory variants may be very helpful if restricted to candidate genes known to be involved in the patient’s disease. This might also reveal the ‘second mutation’ in case of recessive disorders where likely deleterious variants such as premature termination codons are only found on one allele.

Although FABIAN-variant is in principle capable of analysing all variants found in a typical whole-genome sequencing project, we have reduced the number of variants subjected to analysis to 10 000. Generating millions of results for each of the 1387 transcription factors covered by FABIAN-variant would lead to a plethora of data nobody would or could study. Instead, we provide filter options to restrict the analysis to variants found in a specific region or near functional or positional candidate genes.

A limitation of FABIAN-variant is that larger deletions that abolish the complete TFBS cannot be analysed because our application is aimed at the analysis of variants within the TFBS.

FABIAN-variant is aimed at the fast analysis of the variant effect on transcription factor binding on the sequence level and does not consider regulatory features that might affect transcription factor binding (e.g. chromatin accessibility, as implemented in SEMpl (39)).

## OUTLOOK

The infrastructure of FABIAN-variant has been implemented in a way that is easily extensible when new versions of the underlying data are released. Additionally, we are considering adding deep learning-based models such as DeepBind (40) to the application.

We also plan to provide a simple API for the analysis of single variants from within other applications.

## DATA AVAILABILITY

FABIAN-variant can be accessed at https://www.genecascade.org/fabian/. This website is free and open to all users without login requirement or use of cookies.

The results page for each analysis has a unique URL that can be used to access, share, or download results at a later time. Results are kept on the server for three days, after which time they are automatically deleted. Users can also choose to directly delete their data on the results page.

Links to the documentation and a tutorial are provided on the homepage. The documentation has a link to download the underlying TFFM and PWM model definitions.
